# Body Composition Parameters May Be Prognostic Factors in Upper Urinary Tract Urothelial Carcinoma Treated by Radical Nephroureterectomy

**DOI:** 10.3389/fonc.2021.679158

**Published:** 2021-05-24

**Authors:** Yulong Pan, Zeyu Chen, Lanqing Yang, Xingyuan Wang, Zeng Yi, Liang Zhou, Yongjiang Chen, Lu Yang, Hui Zhuo, Yige Bao, Qiang Wei

**Affiliations:** ^1^ West China Hospital, Sichuan University, Chengdu, China; ^2^ Department of Urology, Chengdu Third People’s Hospital, Chengdu, China

**Keywords:** upper urinary tract urothelial carcinoma, body composition parameters, prognostic factors, radical nephroureterectomy, computed tomography

## Abstract

**Objective:**

This study assessed the association between body composition and prognosis of patients with upper urinary tract urothelial carcinoma (UTUC) patients treated by radical nephroureterectomy.

**Methods:**

We retrospectively collected baseline data on age, sex, body mass index (BMI), hypertension, diabetes, and tumor-related factors. Computed tomography (CT) scans were performed to measure body composition parameters such as muscle attenuation (MA), total abdominal muscle area (TAMA), visceral fat area (VFA), intermuscular fat area (IMF), and lateral/posterior perirenal fat thickness (L/P PNF), visceral fat density (VD), and subcutaneous fat density (SD). Patient follow-up was conducted *via* telephone or in the clinic. The endpoints of follow-up were all-cause death, local progression or distant metastasis. Survival analysis was analyzed using the Kaplan-Meier method, and risk factors associated with prognosis were identified using univariate and multivariate Cox proportional hazard analyses.

**Results:**

Among the 273 UTUC patients (median age, 68 years) enrolled in our study, 102 had a BMI > 24.0, 100 suffered from diabetes, and 120 had hypertension. A large proportion of patients (189) had high grade tumors. Across all patients, 1- and 3-year rates for overall survival were 86.45% and 75.55%; local progression-free survival, 92.11% and 89.67%; and distant metastasis-free survival, 85.23% and 80.17%. Based on the Cox regression analysis, MA, IMF, TAMA, TPA, TPT, APT, SMI and PMI significantly reduced the risk of local progression (p < 0.05), while PPNF = 1 point reduced the risk of distant metastasis (p < 0.05). Overall survival was significantly associated with MA, TAMA, and SMI (p < 0.05).

**Conclusion:**

Our findings illustrate that body composition parameters can act as independent predictors of prognosis in UTUC patients who underwent RNU. These results can help improve stratification of patients and optimize postoperative treatment.

## Introduction

Urothelial cancer is an aggressive disease associated with high recurrence and progression rates (1). Upper urinary tract urothelial carcinoma (UTUC) accounts for approximately 10% of all renal tumors and 5-10% of all urothelial carcinomas; about 60% of UTUCs are invasive at diagnosis (1). The standard treatment for UTUC is radical nephroureterectomy (RNU) along with excision of the bladder cuff (1).

Body mass index (BMI) is an international indicator of body weight and thinness, as well as of an individual’s health status. Studies have reported a strong correlation between BMI and prognosis in tumor patients (2, 3). However, individuals with the same BMI can have distinct body compositions, due to differences in body structure and functioning (4). The human body consists of lean tissue (i.e. muscle tissue), and adipose tissue, which stores fat. BMI is a relatively crude measure that does not take into account the individual effects of lean tissue, bones, and adipose tissue, nor does it describe the distribution of fat in the body (5–7). Studies have reported differences in BMI based on ethnicity (8), which therefore has confounded efforts to examine the association between BMI and prognosis. Additionally, muscles and adipose tissue are increasingly being recognized as separate organs, since they play important, but unique roles in the occurrence, progression, prognosis, and other aspects of diseases (5–7).

Apart from its effect on disease prognosis, body composition is also associated with risk of surgical complications, therapeutic efficacy, and treatment-related side effects (9). In this study, we retrospectively analyzed whether individual body composition parameters, including muscle and adipose tissue, are associated with prognosis of UTUC patients treated by RNU.

## Materials and Methods

### Study Population

We retrospectively collected relevant data on UTUC patients registered in our hospital information system between January 2014 and June 2017. We included patients who received a pathological diagnosis of UTUC and had undergone RNU treatment at our hospital. We excluded patients who showed signs of other malignant neoplasms and those with metastatic tumors at local lymph nodes or distant organs at initial diagnosis. We collected data on baseline and clinical characteristics, including age, sex, weight, height, BMI, body composition parameters (see below), diagnosis of hypertension and diabetes, as well as various tumor characteristics (stage, primary site, laterality, grade, and maximum diameter). Due to the retrospective nature of this study, no information on alcohol intake, diet and exercises could be obtained, except for smoking

This study was approved by the Ethics Committee of our hospital.

### Computed Tomography Scans and Body Composition Measurements

For each patient, results of computed tomography (CT) scan using a 64-slice multi detector CT scanner (Somatom Definition Flash, Siemens AG, Erlangen, Germany) was obtained from the database of our center, and conducted segmentation of the CT data using Volume. We followed previously described methods to measure body composition parameters.

We measured total abdominal muscle area (TAMA), which includes the rectus abdominis, intra‐abdominal oblique, external oblique, transverse abdominis, paraspinal, and psoas muscles. We also measured total psoas muscle area (TPA), visceral fat area (VFA), subcutaneous fat area (SFA), visceral fat density (VD) and subcutaneous fat density (SD). Mean muscle attenuation (MA) and intermuscular fat tissue were measured at the level of the L3 inferior endplate, while the axial/transversal psoas thickness (APT/TPT) and subcutaneous fat thickness (SCF) were measured at the level of the umbilicus. Perirenal fat area (PFA) and lateral/posterior perirenal fat thickness (L/P PNF) were measured at the level of the renal vein (< 1 cm = 0 points, 1.1-1.9 cm = 1 point, > 2 cm = 2 points) (10). Tissue was defined as fat if its CT value fell within a range from -150 to -50 Hounsfield units, or as muscle if its CT value fell within a range from -29 to 150 Hounsfield units (10, 11).

### Data Standardization and End Points of Study

Using data collected on fat and muscle area, we derived height-normalized indices (reported as cm^2^/m^2^) for subcutaneous fat (SFI), intermuscular fat (IFI), visceral fat (VFI), skeletal muscle (SMI), and psoas muscle (PMI) using data collected on fat and muscle area. Similarly, psoas muscle thickness, expressed as standardized APT and TPT, was normalized to height and reported as mm/m.

Based on previous literature, we calculated a VFA to SFA ratio (V/S) to provide a single measure of abdominal fat: an elevated V/S indicated higher visceral fat than subcutaneous fat content, and a V/S ≥ 0.4 was used to define visceral obesity (12). The endpoints of our study were overall survival (OS), defined as all-cause death; local progression (local metastasis or recurrence); and distant metastasis.

### Patients Follow up

Follow-up was conducted based on the risk stratification of patients using EAU guidelines (1). Cystoscopy was conducted for low-risk patients at three months after RNU. If no reccurence was found in the bladder, subsequent cystoscopy was performed after one year and repeated annually. High-risk patients underwent cystoscopy and cytology every three months for the first two years, then every six months thereafter until five years, when checkups were done annually. In the case of high-risk patients, CT urograms and chest CTs were performed every six months for two years, then annually.

### Statistical Analyses

All statistical analyses were performed using Empower 2.0. Differences in baseline characteristics were assessed using a chi-squared test. Continuous data were presented as median (min-max) and categorical data as frequency (%).Patient survival was analyzed using the Kaplan-Meier method. Further, a univariate Cox proportional hazard model was used to analyze OS, local progression-free survival (LPFS), and distant metastasis-free survival (DMFS). After adjusting for tumor stage, grade, tumor size (maximum diameter cut off at 3cm) and lymph node invasion, we included all significant variables (p < 0.05) in a multi-variate Cox proportional hazard analysis.

## Results

### Baseline Characteristics

Among the 273 UTUC patients enrolled in this study, who were aged 30 to 92 years (median 68), 102 patients were considered as overweight (BMI > 24). Many of these patients also suffered from diabetes (n = 100) and hypertension (n = 120). A large proportion of these patients had tumors diagnosed as high-grade based on pathology (n = 189), including 90 patients with T3 tumors and 27 with T4 tumors. Further details on baseline characteristics are provided in **Table 1**.

**Table 1:** Baseline characteristics of study cohort.

	Median (Min-Max)
Age	68.00 (30.00-92.00)
Muscle attenuation(MA)	30.70 (1.20-57.10)
IMF(intermuscular)	5.14 (0.62-17.65)
TAMA	53.87 (12.81-104.12)
TPA	8.50 (2.18-42.44)
VFA	58.48 (6.52-155.53)
SFA	55.09 (6.27-199.02)
Abdominal wall fat thickness	1.83 (0.31-4.43)
TPT	2.87 (1.45-5.56)
APT	3.90 (1.94-5.43)
LPNF	1.68 (0.10-5.05)
PPNF	1.17 (0.10-4.43)
PFA	7.51 (0.21-29.30)
SMI	20.63 (5.69-38.24)
PMI	3.24 (0.92-13.86)
SFA(standardized)	21.09 (2.30-70.51)
TPT(standardized)	2.39 (0.79-2570.18)
APT(standardized)	2.41 (1.21-3.28)
VFA/SFA	1.05 (0.22-2.89)
VFA(standardized)	22.84 (2.49-60.00)
(VFA+SFA+IFA)/TAMA	2.46 (0.24-6.34)
VD(visceral fat density)	-96.30 (-116.10–74.30)
SD(subcutaneous fat density)	-102.60 (-124.40–72.70)
	N (%)
Gender	
Male	148 (54.21%)
Female	125 (45.79%)
BMI	
<18.5	29 (10.78%)
18.5-23.9	138 (51.30%)
24-26.9	64 (23.79%)
27-29.9	35 (13.01%)
≥29.9	3 (1.12%)
IFG(≥ 5.6)or type 2 diabetes mellitus	
Yes	169 (62.83%)
No	100 (37.17%)
Hypertension(≥130/85mmHg)	
No	153 (56.04%)
Yes	120 (43.96%)
Hydronephrosis	
No	110 (41.35%)
Yes	156 (58.65%)
Primary site	
Renal pelvis	124 (45.59%)
Ureter	98 (36.03%)
T stage	
Tx, Ta, Tis	8 (2.96%)
T1	95 (35.19%)
T2	50 (18.52%)
T3	90 (33.33%)
T4	27 (10.00%)
Grade	
Low	80 (29.74%)
High	189 (70.26%)
Tumor size(maximum diameter)	
<3cm	82 (30.37%)
≥3cm	188 (69.63%)
Lymph-node invasion	
Without	252 (93.68%)
Identified	17 (6.32%)
Adjuvant therapy	
No	178 (77.06%)
Yes	53 (22.94%)
Laterality	
Left	145 (53.11%)
Right	128 (46.89%)
LPNF(categorized)	
0	86 (40.95%)
1	82 (39.05%)
2	42 (20.00%)
PPNF(categorized)	
0	77 (36.84%)
1	95 (45.45%)
2	37 (17.70%)

### Patient Survival

**Figure 1:**
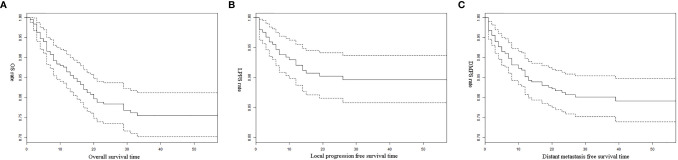
**(A)** Overall survival of all patients; **(B)** Local progression free survival of all patients; **(C)** Distant metastasis free survival of all patients.

During follow-up, we found that 24 (9.56%) patients experienced local progression after RNU, 48 (19.13%) suffered distant metastasis, and 59 (23.51%) died. Kaplan-Meier analysis indicated OS rates of 86.45% at 1 year and 75.55% at 3 years (**Figure 1A**). The corresponding LPFS rates 92.11% and 89.67% (**Figure 1B**), while DMFS rates were 85.23% and 80.17% (**Figure 1C**).

### Prognostic Factors

The uni-variate Cox proportional hazard analyses showed that higher values of MA, TAMA, TPT, SMI, and standardized TPT were associated with lower OS in patients (p < 0.1; **Table 2**). An increase in PMI, MA, TAMA, TPA, TPT, or APT was associated with lower incidence of local progression, while MA, TPA, PPNF =1 point, PMI, and standardized TPT was negatively associated with risk of distant metastasis.

**Table 2:** Univariate cox proportional hazard analysis for risk factors of patients’ prognosis.

	OS	LPS	DM
	HR (95%CI) p-value	HR (95%CI) p-value	HR (95%CI) p-value
MA	0.96 (0.93, 0.98) 0.0015	0.96 (0.92, 1.00) 0.0587	0.96 (0.93, 0.99) 0.0051
IMF	1.05 (0.96, 1.14) 0.2946	0.88 (0.73, 1.06) 0.1638	1.02 (0.93, 1.12) 0.6566
TAMA	0.98 (0.97, 1.00) 0.0556	0.97 (0.95, 1.00) 0.0718	0.99 (0.97, 1.01) 0.2523
TPA	0.96 (0.90, 1.02) 0.1815	0.81 (0.69, 0.96) 0.0161	0.92 (0.84, 1.00) 0.0454
VFA	1.00 (0.99, 1.01) 0.7856	0.99 (0.98, 1.01) 0.3959	1.00 (0.99, 1.01) 0.6543
SFA	1.00 (0.99, 1.01) 0.5291	1.00 (0.98, 1.01) 0.5658	1.00 (0.99, 1.01) 0.7772
Abdominal wall fat thickness	1.23 (0.85, 1.78) 0.2781	1.31 (0.69, 2.49) 0.4015	1.03 (0.68, 1.57) 0.8775
TPT	0.62 (0.40, 0.96) 0.0339	0.36 (0.16, 0.83) 0.0162	0.84 (0.54, 1.32) 0.4491
APT	0.94 (0.61, 1.46) 0.7910	0.50 (0.24, 1.03) 0.0593	0.96 (0.60, 1.53) 0.8704
LPNF	0.94 (0.66, 1.33) 0.7189	0.78 (0.41, 1.49) 0.4598	0.99 (0.69, 1.42) 0.9478
PPNF	1.04 (0.69, 1.56) 0.8514	0.82 (0.38, 1.74) 0.5971	1.10 (0.72, 1.68) 0.6518
PFA	0.98 (0.92, 1.04) 0.4224	0.95 (0.86, 1.06) 0.3808	0.99 (0.93, 1.05) 0.7929
LPNF(categorized)			
0	1.0	1.0	1.0
1	0.83 (0.44, 1.57) 0.5641	0.63 (0.21, 1.93) 0.4188	0.71 (0.35, 1.43) 0.3365
2	0.75 (0.33, 1.69) 0.4862	0.48 (0.10, 2.26) 0.3528	0.94 (0.43, 2.08) 0.8848
PPNF(categorized)			
0	1.0	1.0	1.0
1	0.65 (0.34, 1.21) 0.1726	0.47 (0.15, 1.44) 0.1885	0.31 (0.14, 0.68) 0.0034
2	0.65 (0.27, 1.52) 0.3155	0.48 (0.10, 2.24) 0.3468	0.91 (0.43, 1.94) 0.8161
SMI	0.96 (0.91, 1.01) 0.0932	0.94 (0.86, 1.02) 0.1187	0.97 (0.92, 1.03) 0.3063
PMI	0.90 (0.76, 1.06) 0.2090	0.57 (0.36, 0.90) 0.0169	0.79 (0.62, 1.00) 0.0507
SFA(standardized)	1.00 (0.97, 1.02) 0.7228	0.99 (0.96, 1.04) 0.7899	1.00 (0.98, 1.03) 0.6957
TPT(standardized)	0.56 (0.29, 1.06) 0.0765	0.56 (0.17, 1.80) 0.3275	0.55 (0.28, 1.08) 0.0810
APT(standardized)	1.15 (0.51, 2.57) 0.7417	0.36 (0.10, 1.28) 0.1147	1.03 (0.44, 2.40) 0.9418
VFA/SFA	0.98 (0.53, 1.81) 0.9443	0.72 (0.24, 2.14) 0.5531	0.84 (0.42, 1.69) 0.6321
VFA(standardized)	1.00 (0.98, 1.02) 0.9998	0.99 (0.95, 1.03) 0.6399	1.01 (0.98, 1.03) 0.5012
(VFA+SFA+IFA)/TAMA	1.11 (0.88, 1.39) 0.3859	1.13 (0.76, 1.66) 0.5503	1.15 (0.90, 1.47) 0.2521
VD	1.02 (0.99, 1.05) 0.2256	1.03 (0.98, 1.09) 0.2880	1.00 (0.96, 1.04) 0.9838
SD	1.00 (0.97, 1.03) 0.8808	1.02 (0.97, 1.07) 0.3878	0.99 (0.96, 1.03) 0.6732

After adjusting for confounding factors, the results of the adjusted model analysis showed that there was a significant association between MA, TAMA, SMI and OS (P<0.05). Additionally, MA, IMF, TAMA, TPA, TPT, APT, SMI and PMI were found to be inversely related to the risk of local progression, while PPNF = 1 point was associated with significantly lower risk of distant metastasis (**Table 3**).

**Table 3:** Multivariate cox proportional hazard analysis for risk factors of patients’ prognosis.

	OS	LPS	DM
	HR (95%CI) p-value	HR (95%CI) p-value	HR (95%CI) p-value
MA	0.96 (0.94, 0.99) 0.0108	0.97 (0.94, 1.00) 0.0334	0.97 (0.94, 1.00) 0.0743
IMF	1.01 (0.91, 1.12) 0.8035	0.76 (0.58, 0.98) 0.0344	0.95 (0.84, 1.07) 0.3918
TAMA	0.97 (0.95, 0.99) 0.0065	0.96 (0.92, 0.99) 0.0155	0.98 (0.96, 1.00) 0.0603
TPA	0.97 (0.91, 1.04) 0.4234	0.75 (0.61, 0.92) 0.0059	0.92 (0.83, 1.02) 0.1159
TPT	0.78 (0.50, 1.22) 0.2757	0.35 (0.14, 0.85) 0.0206	0.95 (0.58, 1.55) 0.8379
APT	0.97 (0.59, 1.62) 0.9183	0.39 (0.16, 0.95) 0.0374	1.03 (0.59, 1.81) 0.9178
PPNF			
0	1.0	1.0	1.0
1	0.80 (0.41, 1.54) 0.5046	0.56 (0.18, 1.76) 0.3231	0.31 (0.13, 0.73) 0.0079
2	0.58 (0.23, 1.46) 0.2450	0.27 (0.03, 2.22) 0.2253	0.88 (0.38, 2.02) 0.7555
SMI	0.93 (0.87, 0.98) 0.0143	0.89 (0.80, 0.99) 0.0252	0.94 (0.88, 1.01) 0.0798
PMI	0.94 (0.79, 1.13) 0.5313	0.45 (0.26, 0.79) 0.0053	0.81 (0.60, 1.08) 0.1437

## Discussion

Although both UTUC and bladder cancer involve malignant tumors in the urinary epithelium, UTUC has typically invaded the muscle layer by the time it is diagnosed. Therefore, UTUC patients have a significantly worse prognosis than those with bladder cancer, and they remain at high risk of tumor recurrence and metastasis even after RNU (1). Few studies have examined the combined prognostic effect of muscle and fat tissues, particularly in the Chinese population. To our knowledge, this is the first study examining the relationship of muscle and fat-related parameters with survival outcomes in UTUC patients treated with RNU. We found that muscle quality, muscle quantity, and perirenal fat thickness correlated significantly with survival of UTUC patients after RNU.

Studies involving UTUC patients have typically focused on various factors affecting disease prognosis, including tumor-related factors such as pathology stage and grade, or clinical factors such as physical status and smoking history (1). Age, tumor grade, location, T stage, growth pattern, lymphovascular invasion, and histological variation can affect the prognosis of UTUC patients treated with RNU (1). However, these factors depend on postoperative pathological examinations. Few large studies have examined preoperative factors that may help predict prognosis of UTUC patients.

Fat and muscle are two important components of the body that play distinct, yet significant roles in the management of diseases (2, 6). Clinical imaging has proved to be reliable for the assessment of the quantity and distribution of muscle and fat in the body (13, 14). The analysis of CT scans at the level of the third lumbar spine vertebra is considered the gold standard for measuring body composition parameters in muscle and adipose tissue. Among cancer patients undergoing chemotherapy, molecular-targeted therapy or immunotherapy (3, 9, 15), the loss of muscle mass or quality has been associated with decreased OS, increased incidence of surgical complications, as well as increased frequency and severity of adverse reactions. The prognostic effects of adipose tissue parameters vary based on the tumor type (2, 6).

Body composition parameters associated with skeletal mass (SMI and MA) have been identified as prognostic factors in lung cancer, esophageal cancer, gastric cancer, and pancreatic cancer (3, 9, 15, 16). MA is used to measure muscle quality, while SMI is used to measure body muscle mass (17, 18). However, it is unclear whether muscle mass (SMI) or quality (MA) is more suitable to understand disease prognosis in tumor patients (18). For example, in patients with non-small cell lung cancer and pancreatic cancer, only MA is a prognostic factor for OS. Low MA is associated with obesity, diabetes, loss of exercise and muscle atrophy. Studies have found that the attenuation density of skeletal muscle positively correlates with muscle quality and strength, and negatively correlates with muscle fiber fat content. Indeed, attenuation density of skeletal muscle varies inversely with the lipid drop content in muscle fibers (19). The mechanisms underlying these changes in muscle appear to affect its quality earlier, or more severely, than its quantity.

Based on the observed effects of muscle mass and quantity on prognosis, preoperative targeted intervention of muscle state may be an effective way to improve outcomes. Our results argue for preoperative efforts to improve muscle mass of UTUC patients. Studies have reported that resistance training alone does not significantly improve musclemass (17). Further studies are necessary to identify approaches, perhaps combining exercise, nutrition, and drugs, that can effectively build muscle mass in UTUC patients.

Based on previous studies, we analyzed the prognostic effects of psoas muscle parameters (PMI and standardized TPT) on UTUC patients treated with RNU (20, 21). Patients with higher MA, TAMA and SMI values tended to be at lower risk of all-cause death. MA, IMF, TAMA, TPA, TPT, APT, SMI and PMI were associated with a reduction in local progression of the tumor; PPNF = 1 point was also associated with a reduced risk of distant metastasis. Our findings support the idea that psoas characteristics serve as markers of sarcopenia (20, 21).

Studies have reported that fat parameters (VFA, SFA, SCF, IFA, SD, VD) measured at the third lumbar vertebra were associated with prognosis of patients with adenoma-derived tumors, including renal cancer, colorectal cancer, or gastric carcinoma (11, 22). We found that these parameters had no effect on the prognosis of UTUC patients. This can be due to the small sample size in our study, or the differences between urothelial carcinoma and adenocarcinoma at the cellular level: adenocarcinoma cells contain large amounts of lipid droplets, while urothelial carcinoma cells do not, and so lipid metabolism may not play a significant role in the latter cancer. Meanwhile, our results also suggest that perirenal or periureteral adipose tissue may significantly influence urothelial carcinoma invasion and progression.

Our results must be interpreted with caution in the light of certain limitations. The retrospective design of the study and the relatively small cohort are potential sources of bias. It was impossible to retrospectively obtain data on important diagnostic factors associated with sarcopenia, such as grip strength, walking speed, and patient self‐report of the SARC-F questionnaire (23). Additionally, we did not have sufficient imaging data to compare changes in body composition before and after RNU treatment. Future work should focus on validating and extending these results.

## Conclusion

Our findings show that preoperative assessment of body condition that is more finely grained than BMI can help predict prognosis of UTUC patients after RNU. We found that body composition parameters associated with muscle quality and muscle mass, can independently predict prognosis in such patients. Our results may help improve risk stratification of patients and their postoperative management.

## Data Availability Statement

The raw data supporting the conclusions of this article will be made available by the authors, without undue reservation.

## Ethics Statement

The studies involving human participants were reviewed and approved by Ethics Committee on Biomedical Research, West China Hospital of Sichuan University. The patients/participants provided their written informed consent to participate in this study.

## Author Contributions

YP: data acquisition, investigation, writing - original draft, and formal analysis. ZC: conceptualization, data curation, formal analysis, software, validation, and writing - review & editing. LaY: software, visualization, and data acquisition. XW: data curation and software. ZY: formal analysis and investigation. LZ: visualization and data acquisition. YC: Writing - review & editing. LuY: software, investigation, and supervision. HZ: investigation and methodology. YB: supervision, validation, and project administration. QW: supervision, project administration, funding acquisition. All authors contributed to the article and approved the submitted version.

## Funding

This work was supported by the National Natural Science Foundation of China (Grant number: 81500522) and Science & Technology Department of Sichuan Province (Grant number: 2020YFS0090, 2020YFS0046).

## Conflict of Interest

The authors declare that the research was conducted in the absence of any commercial or financial relationships that could be construed as a potential conflict of interest.
