# Erratum: Body Composition Parameters May Be Prognostic Factors in Upper Urinary Tract Urothelial Carcinoma Treated by Radical Nephroureterectomy

**DOI:** 10.3389/fonc.2021.740572

**Published:** 2021-07-23

**Authors:** Frontiers Production Office

**Affiliations:** Frontiers Media SA, Lausanne, Switzerland

**Keywords:** upper urinary tract urothelial carcinoma, body composition parameters, prognostic factors, radical nephroureterectomy, computed tomography

Due to a production error, the equal contribution of Zeyu Chen and Lanqing Yang was not included. The publisher apologizes for this mistake.

